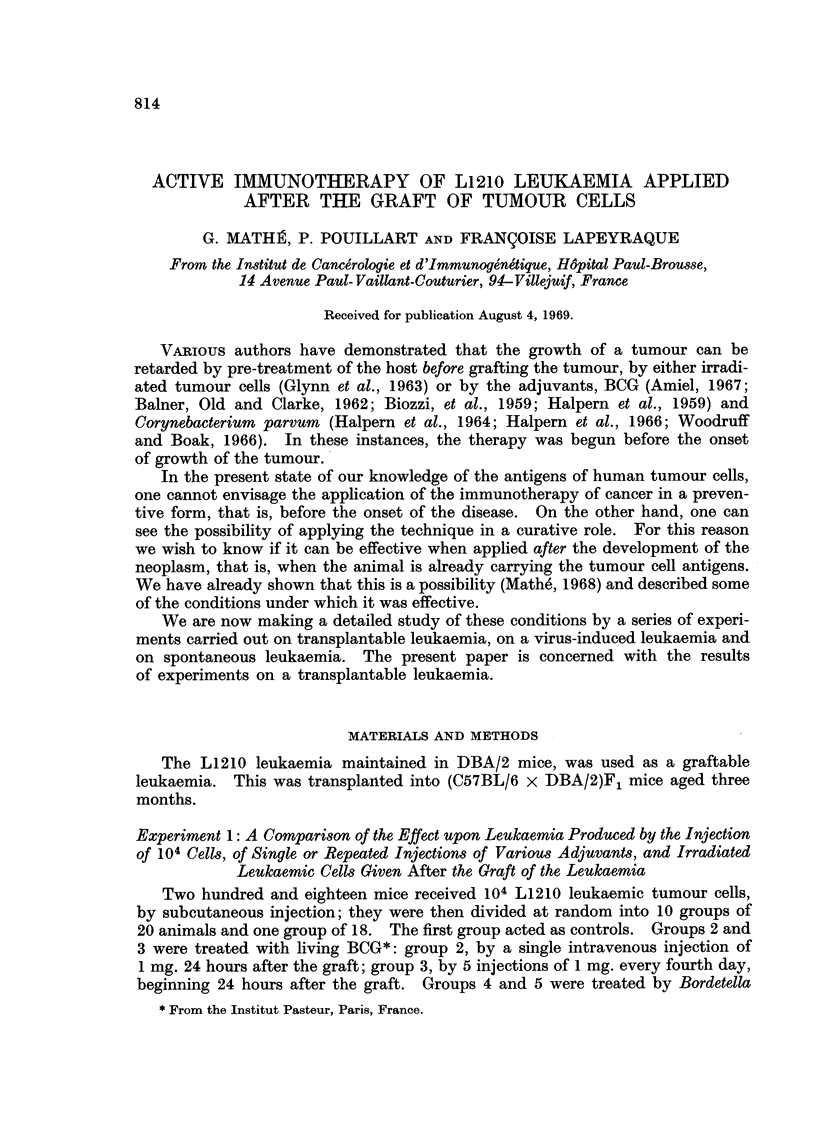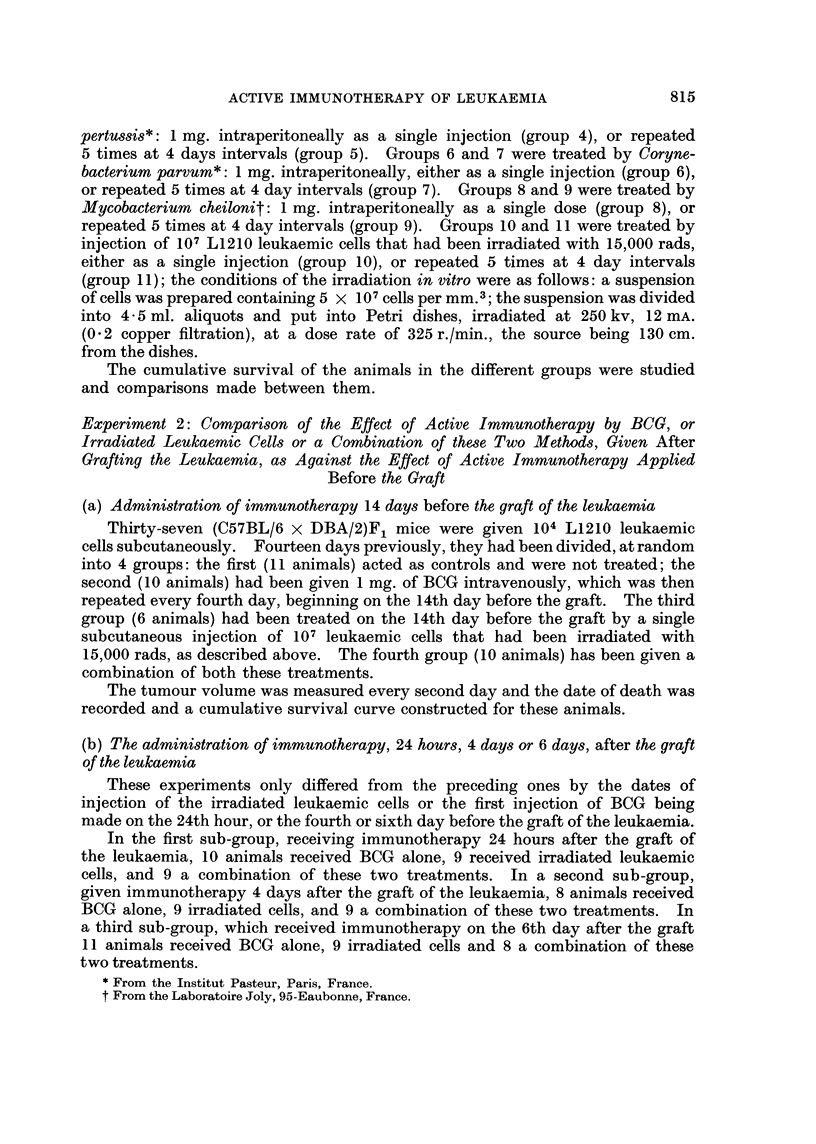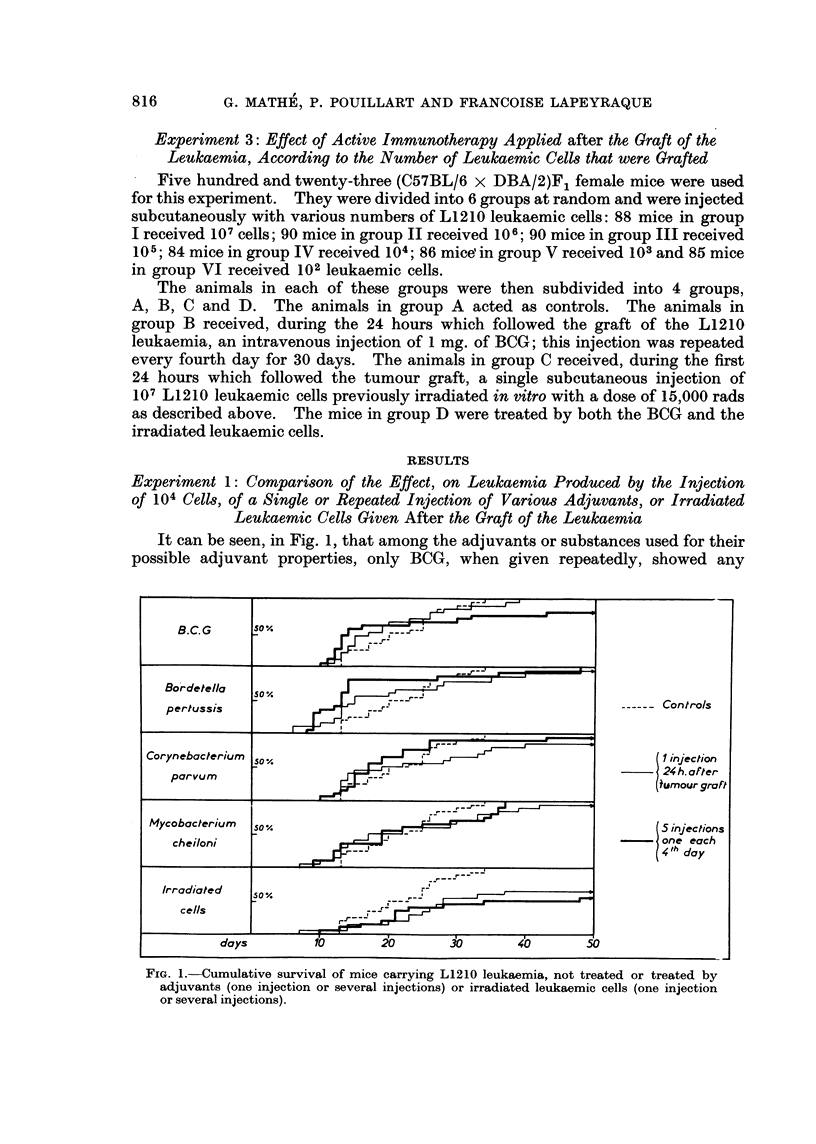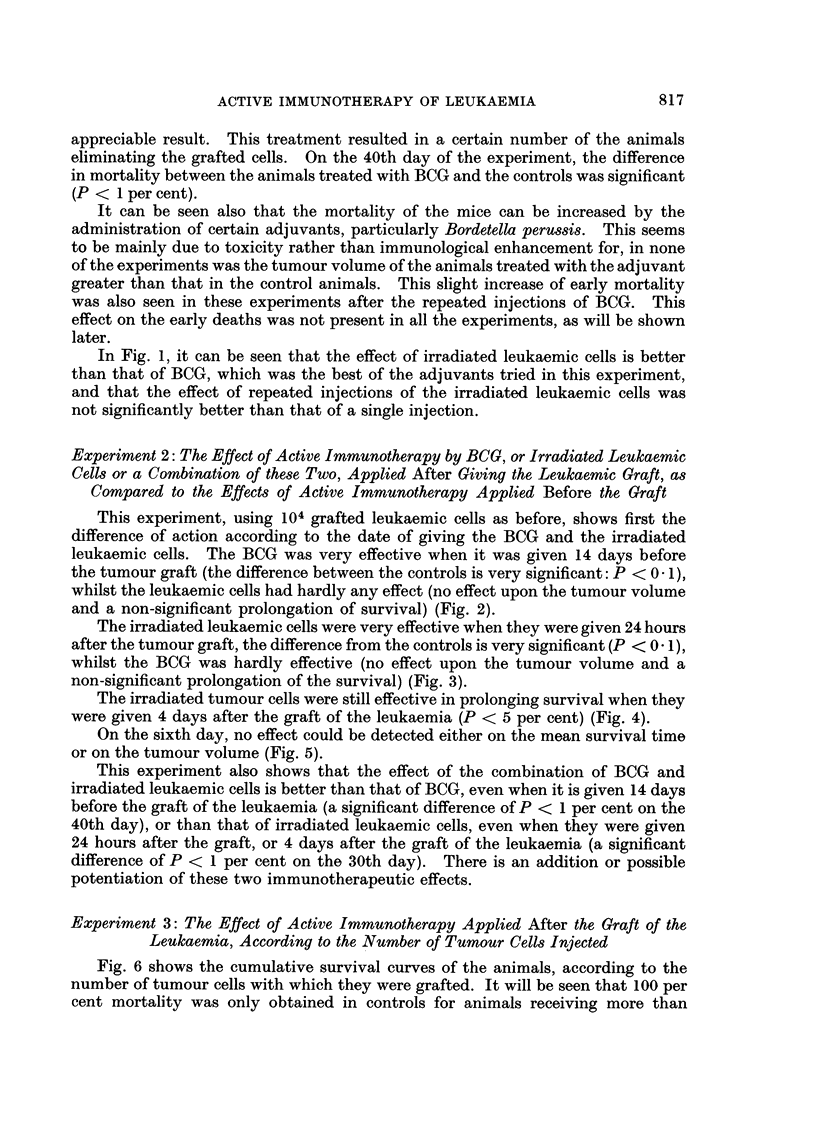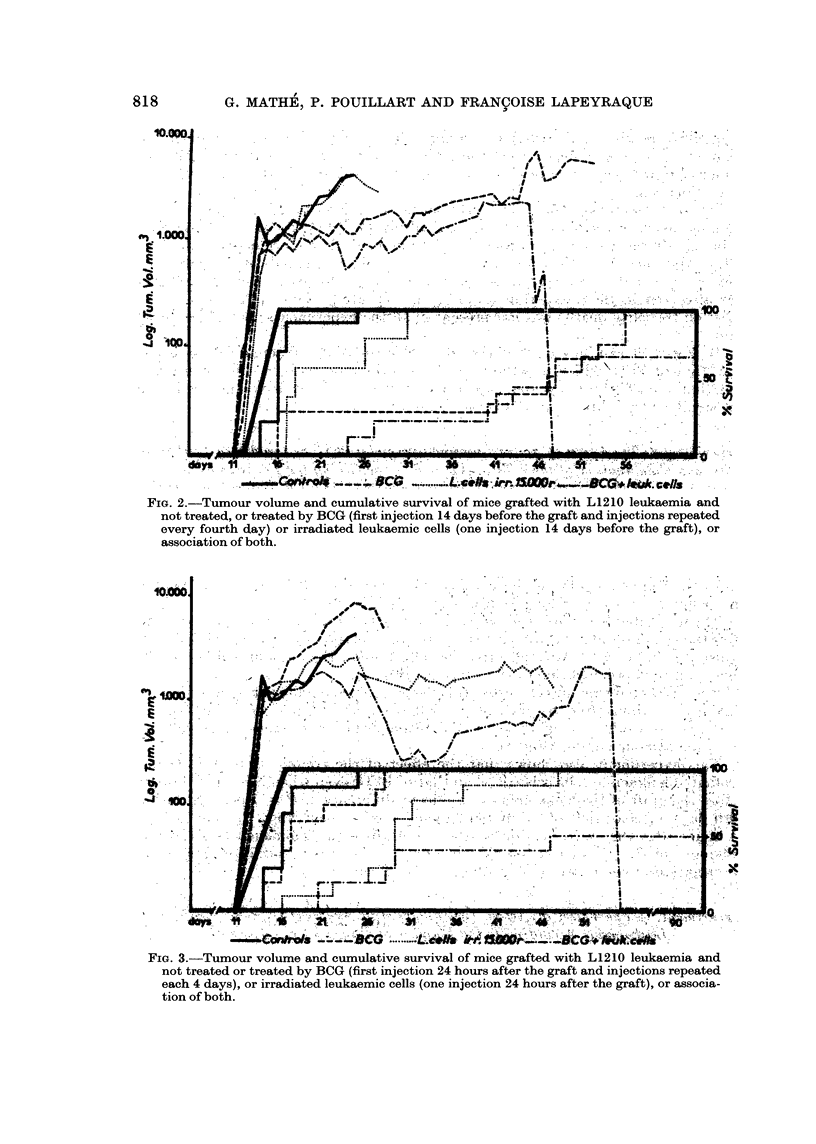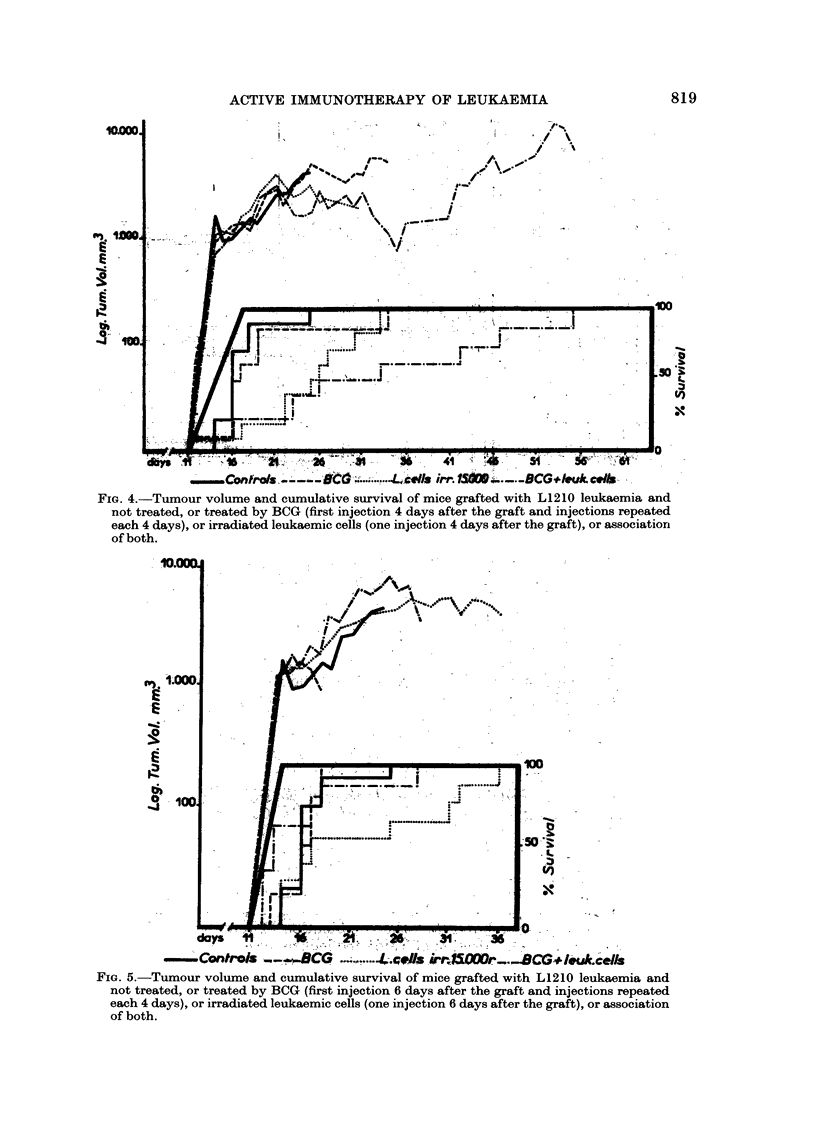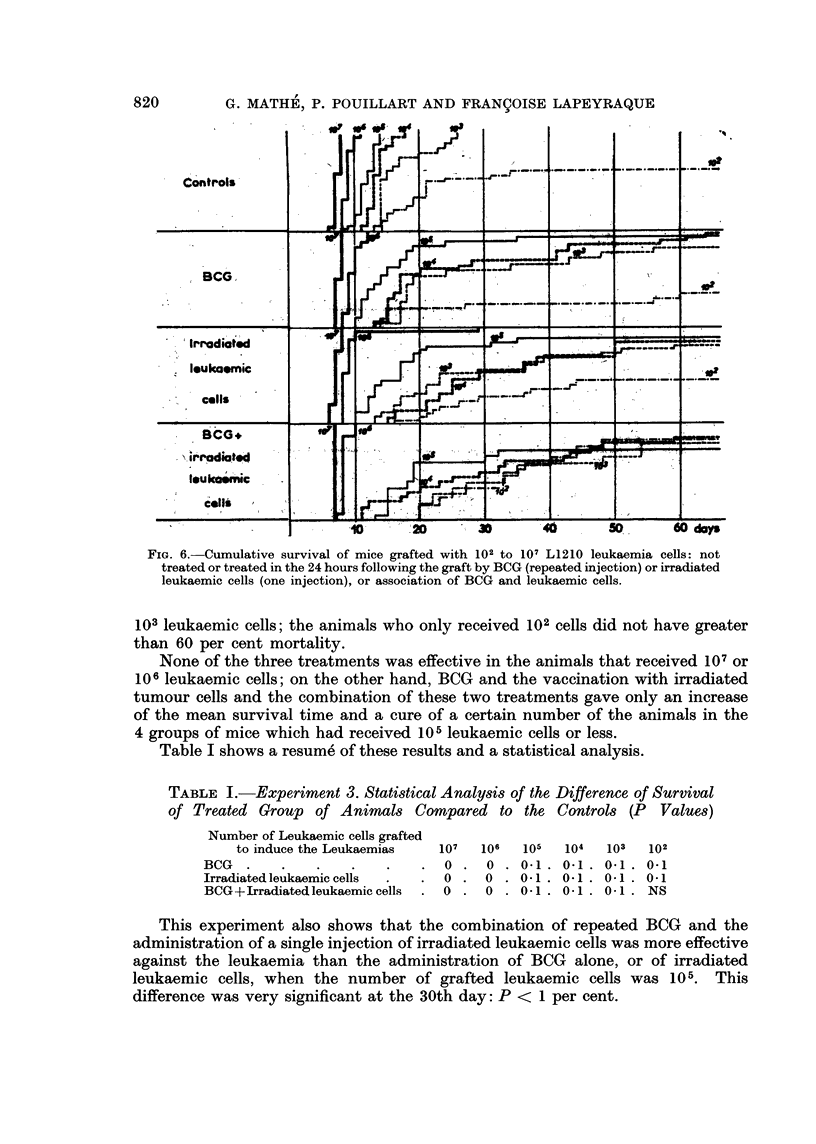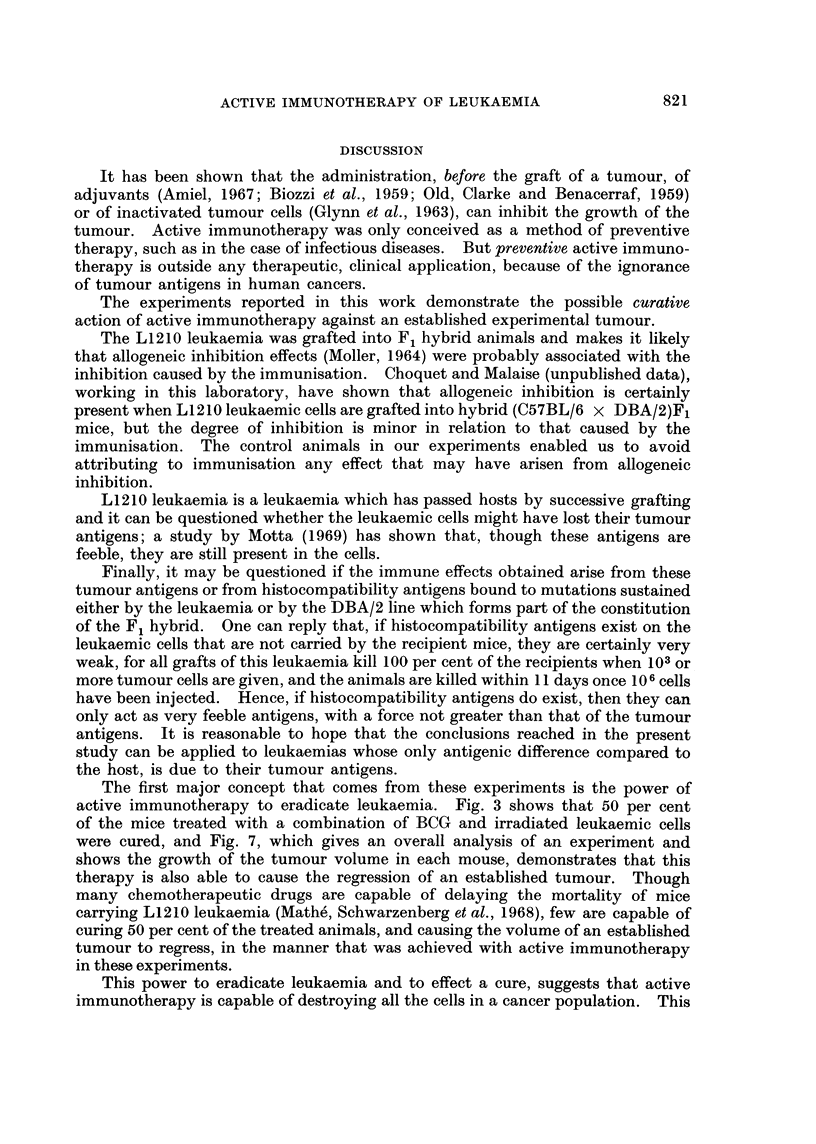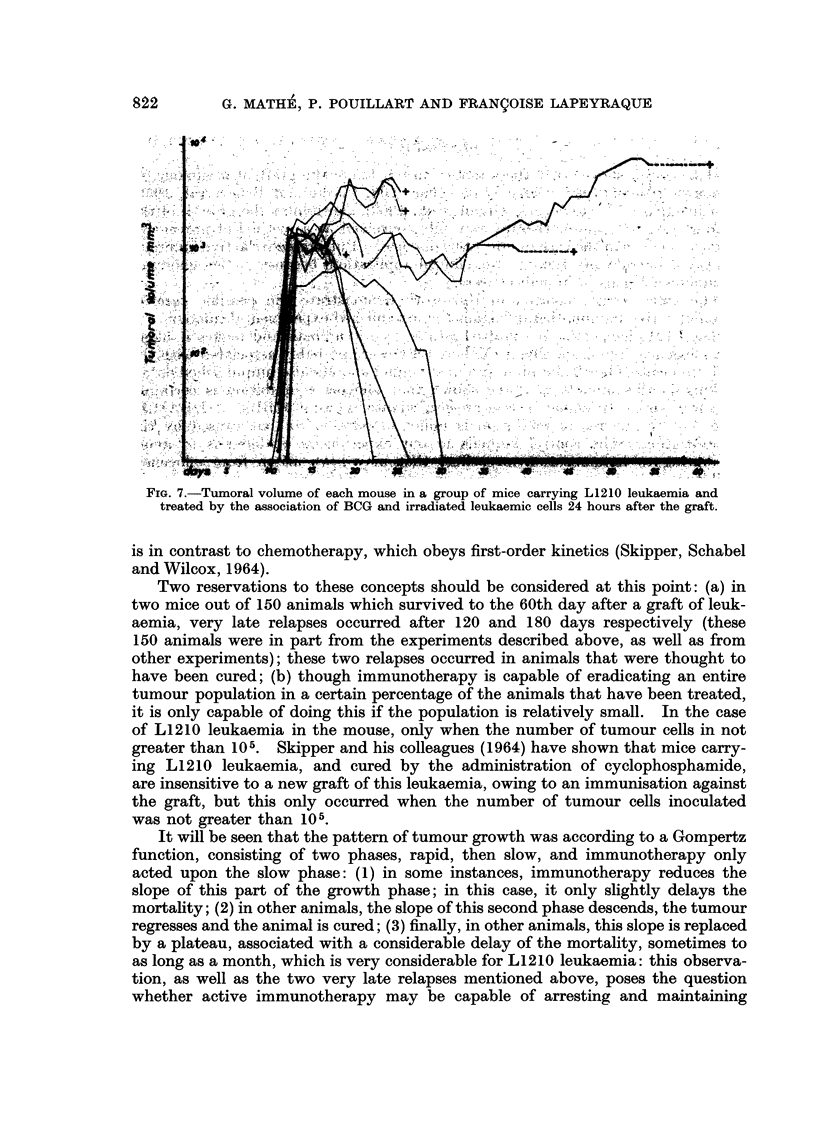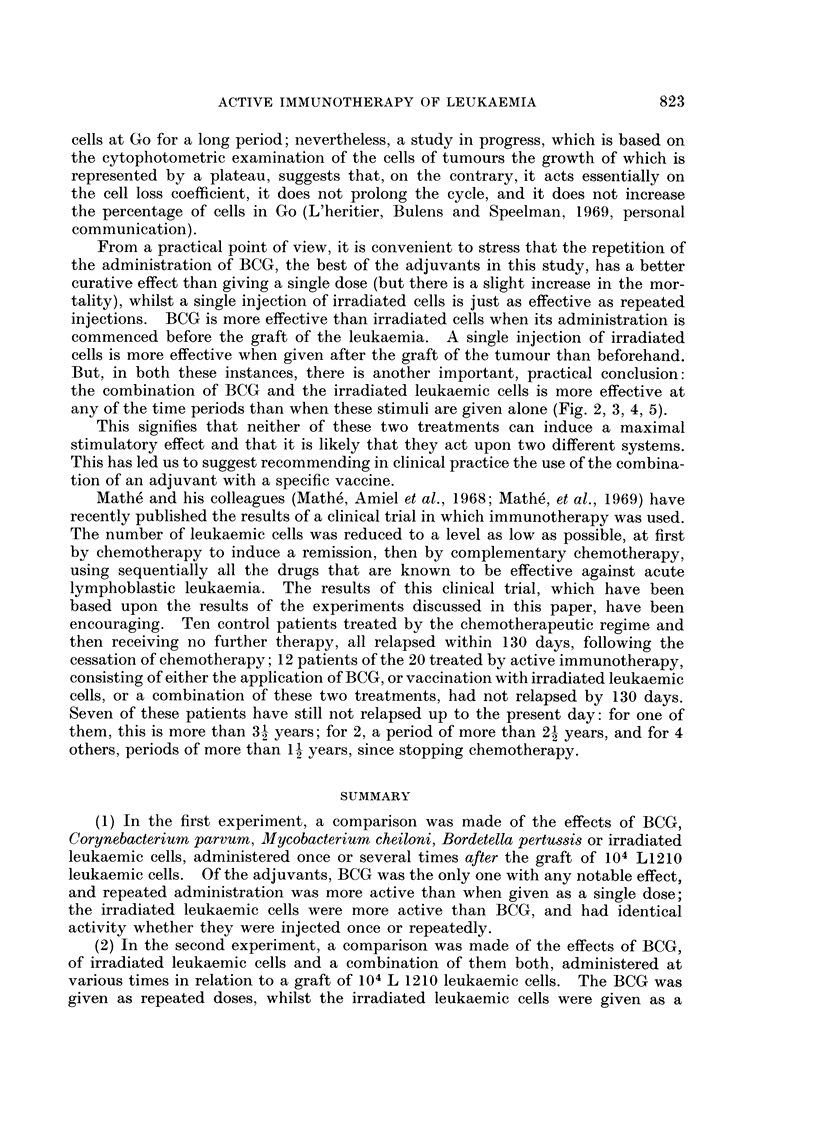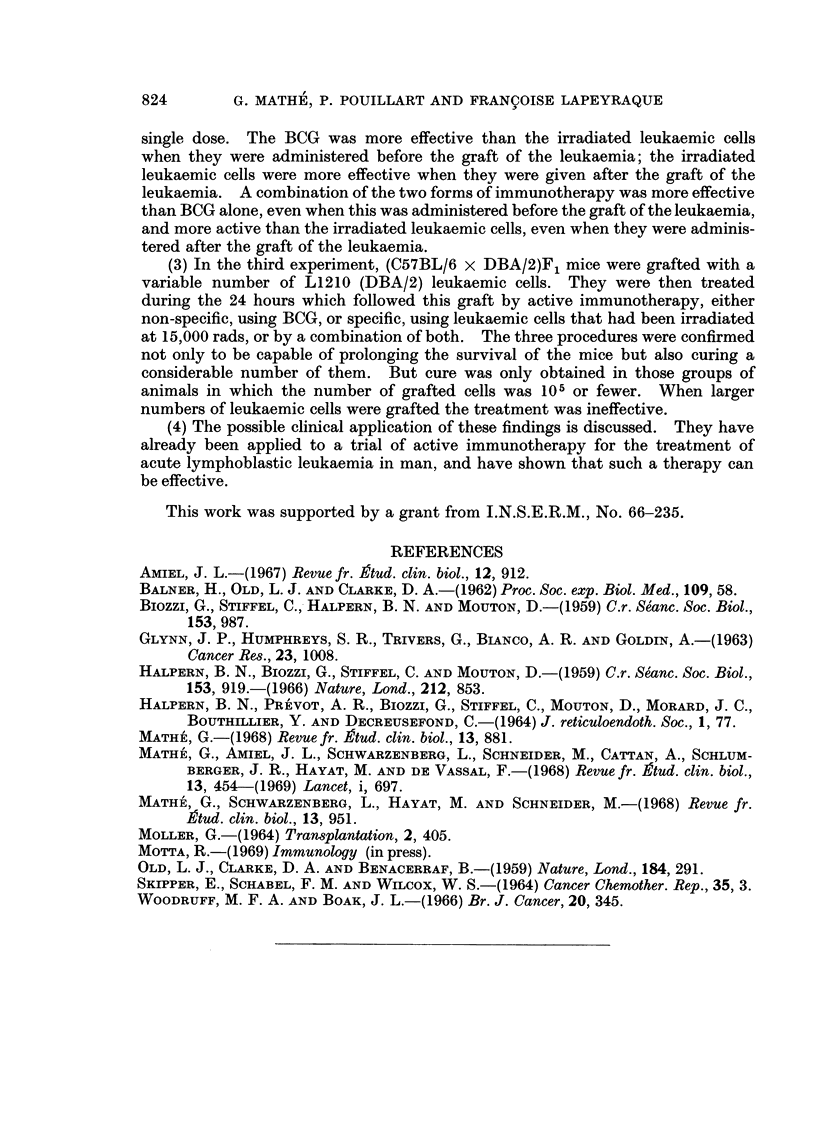# Active immunotherapy of L1210 leukaemia applied after the graft of tumour cells.

**DOI:** 10.1038/bjc.1969.101

**Published:** 1969-12

**Authors:** G. Mathé, P. Pouillart, F. Lapeyraque


### ACTIVE IMMUNOTHERAPY OF L1210 LEUKAEMIA APPLIED AFTER THE GRAFT OF TUMOUR CELLS

G. MATHÉ, P. POUILLART AND FRANÇOISE LAPEYRAQUE

*From the Institut de Cancérologie et d'Immunogénétique, Hôpital Paul-Brousse, 14 Avenue Paul-Vaillant-Couturier, 94–Villejuif, France*

Received for publication August 4, 1969.

VARIOUS authors have demonstrated that the growth of a tumour can be retarded by pre-treatment of the host *before* grafting the tumour, by either irradiated tumour cells (Glynn *et al.*, 1963) or by the adjuvants, BCG (Amiel, 1967; Balner, Old and Clarke, 1962; Biozzi, *et al.*, 1959; Halpern *et al.*, 1959) and *Corynebacterium parvum* (Halpern *et al.*, 1964; Halpern *et al.*, 1966; Woodruff and Boak, 1966). In these instances, the therapy was begun before the onset of growth of the tumour.

In the present state of our knowledge of the antigens of human tumour cells, one cannot envisage the application of the immunotherapy of cancer in a preventive form, that is, before the onset of the disease. On the other hand, one can see the possibility of applying the technique in a curative role. For this reason we wish to know if it can be effective when applied *after* the development of the neoplasm, that is, when the animal is already carrying the tumour cell antigens. We have already shown that this is a possibility (Mathé, 1968) and described some of the conditions under which it was effective.

We are now making a detailed study of these conditions by a series of experiments carried out on transplantable leukaemia, on a virus-induced leukaemia and on spontaneous leukaemia. The present paper is concerned with the results of experiments on a transplantable leukaemia.

### MATERIALS AND METHODS

The L1210 leukaemia maintained in DBA/2 mice, was used as a graftable leukaemia. This was transplanted into (C57BL/6 × DBA/2)$F_1$ mice aged three months.

### *Experiment* 1: *A Comparison of the Effect upon Leukaemia Produced by the Injection of* $10^4$ *Cells, of Single or Repeated Injections of Various Adjuvants, and Irradiated Leukaemic Cells Given* After *the Graft of the Leukaemia*

Two hundred and eighteen mice received $10^4$ L1210 leukaemic tumour cells, by subcutaneous injection; they were then divided at random into 10 groups of 20 animals and one group of 18. The first group acted as controls. Groups 2 and 3 were treated with living BCG*: group 2, by a single intravenous injection of 1 mg. 24 hours after the graft; group 3, by 5 injections of 1 mg. every fourth day, beginning 24 hours after the graft. Groups 4 and 5 were treated by *Bordetella*

* From the Institut Pasteur, Paris, France.

*pertussis**: 1 mg. intraperitoneally as a single injection (group 4), or repeated 5 times at 4 days intervals (group 5). Groups 6 and 7 were treated by *Corynebacterium parvum**: 1 mg. intraperitoneally, either as a single injection (group 6), or repeated 5 times at 4 day intervals (group 7). Groups 8 and 9 were treated by *Mycobacterium cheiloni*†: 1 mg. intraperitoneally as a single dose (group 8), or repeated 5 times at 4 day intervals (group 9). Groups 10 and 11 were treated by injection of $10^7$ L1210 leukaemic cells that had been irradiated with 15,000 rads, either as a single injection (group 10), or repeated 5 times at 4 day intervals (group 11); the conditions of the irradiation *in vitro* were as follows: a suspension of cells was prepared containing $5 \times 10^7$ cells per mm.$^3$; the suspension was divided into 4·5 ml. aliquots and put into Petri dishes, irradiated at 250 kv, 12 mA. (0·2 copper filtration), at a dose rate of 325 r./min., the source being 130 cm. from the dishes.

The cumulative survival of the animals in the different groups were studied and comparisons made between them.

*Experiment 2: Comparison of the Effect of Active Immunotherapy by BCG, or Irradiated Leukaemic Cells or a Combination of these Two Methods, Given* After *Grafting the Leukaemia, as Against the Effect of Active Immunotherapy Applied* Before *the Graft*

(a) *Administration of immunotherapy* 14 *days* before *the graft of the leukaemia*

Thirty-seven (C57BL/6 × DBA/2)$F_1$ mice were given $10^4$ L1210 leukaemic cells subcutaneously. Fourteen days previously, they had been divided, at random into 4 groups: the first (11 animals) acted as controls and were not treated; the second (10 animals) had been given 1 mg. of BCG intravenously, which was then repeated every fourth day, beginning on the 14th day before the graft. The third group (6 animals) had been treated on the 14th day before the graft by a single subcutaneous injection of $10^7$ leukaemic cells that had been irradiated with 15,000 rads, as described above. The fourth group (10 animals) has been given a combination of both these treatments.

The tumour volume was measured every second day and the date of death was recorded and a cumulative survival curve constructed for these animals.

(b) *The administration of immunotherapy*, 24 *hours*, 4 *days or* 6 *days*, after *the graft of the leukaemia*

These experiments only differed from the preceding ones by the dates of injection of the irradiated leukaemic cells or the first injection of BCG being made on the 24th hour, or the fourth or sixth day before the graft of the leukaemia.

In the first sub-group, receiving immunotherapy 24 hours after the graft of the leukaemia, 10 animals received BCG alone, 9 received irradiated leukaemic cells, and 9 a combination of these two treatments. In a second sub-group, given immunotherapy 4 days after the graft of the leukaemia, 8 animals received BCG alone, 9 irradiated cells, and 9 a combination of these two treatments. In a third sub-group, which received immunotherapy on the 6th day after the graft 11 animals received BCG alone, 9 irradiated cells and 8 a combination of these two treatments.

* From the Institut Pasteur, Paris, France.
† From the Laboratoire Joly, 95-Eaubonne, France.

*Experiment* 3: *Effect of Active Immunotherapy Applied* after *the Graft of the Leukaemia, According to the Number of Leukaemic Cells that were Grafted*

Five hundred and twenty-three (C57BL/6 × DBA/2)$F_1$ female mice were used for this experiment. They were divided into 6 groups at random and were injected subcutaneously with various numbers of L1210 leukaemic cells: 88 mice in group I received $10^7$ cells; 90 mice in group II received $10^6$; 90 mice in group III received $10^5$; 84 mice in group IV received $10^4$; 86 mice in group V received $10^3$ and 85 mice in group VI received $10^2$ leukaemic cells.

The animals in each of these groups were then subdivided into 4 groups, A, B, C and D. The animals in group A acted as controls. The animals in group B received, during the 24 hours which followed the graft of the L1210 leukaemia, an intravenous injection of 1 mg. of BCG; this injection was repeated every fourth day for 30 days. The animals in group C received, during the first 24 hours which followed the tumour graft, a single subcutaneous injection of $10^7$ L1210 leukaemic cells previously irradiated *in vitro* with a dose of 15,000 rads as described above. The mice in group D were treated by both the BCG and the irradiated leukaemic cells.

### RESULTS

*Experiment* 1: *Comparison of the Effect, on Leukaemia Produced by the Injection of* $10^4$ *Cells, of a Single or Repeated Injection of Various Adjuvants, or Irradiated Leukaemic Cells Given* After *the Graft of the Leukaemia*

It can be seen, in Fig. 1, that among the adjuvants or substances used for their possible adjuvant properties, only BCG, when given repeatedly, showed any

FIG. 1.—Cumulative survival of mice carrying L1210 leukaemia, not treated or treated by adjuvants (one injection or several injections) or irradiated leukaemic cells (one injection or several injections).

appreciable result. This treatment resulted in a certain number of the animals eliminating the grafted cells. On the 40th day of the experiment, the difference in mortality between the animals treated with BCG and the controls was significant ($P < 1$ per cent).

It can be seen also that the mortality of the mice can be increased by the administration of certain adjuvants, particularly *Bordetella perussis*. This seems to be mainly due to toxicity rather than immunological enhancement for, in none of the experiments was the tumour volume of the animals treated with the adjuvant greater than that in the control animals. This slight increase of early mortality was also seen in these experiments after the repeated injections of BCG. This effect on the early deaths was not present in all the experiments, as will be shown later.

In Fig. 1, it can be seen that the effect of irradiated leukaemic cells is better than that of BCG, which was the best of the adjuvants tried in this experiment, and that the effect of repeated injections of the irradiated leukaemic cells was not significantly better than that of a single injection.

*Experiment 2: The Effect of Active Immunotherapy by BCG, or Irradiated Leukaemic Cells or a Combination of these Two, Applied* After *Giving the Leukaemic Graft, as Compared to the Effects of Active Immunotherapy Applied* Before *the Graft*

This experiment, using $10^4$ grafted leukaemic cells as before, shows first the difference of action according to the date of giving the BCG and the irradiated leukaemic cells. The BCG was very effective when it was given 14 days before the tumour graft (the difference between the controls is very significant: $P < 0{\cdot}1$), whilst the leukaemic cells had hardly any effect (no effect upon the tumour volume and a non-significant prolongation of survival) (Fig. 2).

The irradiated leukaemic cells were very effective when they were given 24 hours after the tumour graft, the difference from the controls is very significant ($P < 0{\cdot}1$), whilst the BCG was hardly effective (no effect upon the tumour volume and a non-significant prolongation of the survival) (Fig. 3).

The irradiated tumour cells were still effective in prolonging survival when they were given 4 days after the graft of the leukaemia ($P < 5$ per cent) (Fig. 4).

On the sixth day, no effect could be detected either on the mean survival time or on the tumour volume (Fig. 5).

This experiment also shows that the effect of the combination of BCG and irradiated leukaemic cells is better than that of BCG, even when it is given 14 days before the graft of the leukaemia (a significant difference of $P < 1$ per cent on the 40th day), or than that of irradiated leukaemic cells, even when they were given 24 hours after the graft, or 4 days after the graft of the leukaemia (a significant difference of $P < 1$ per cent on the 30th day). There is an addition or possible potentiation of these two immunotherapeutic effects.

*Experiment 3: The Effect of Active Immunotherapy Applied* After *the Graft of the Leukaemia, According to the Number of Tumour Cells Injected*

Fig. 6 shows the cumulative survival curves of the animals, according to the number of tumour cells with which they were grafted. It will be seen that 100 per cent mortality was only obtained in controls for animals receiving more than

FIG. 2.—Tumour volume and cumulative survival of mice grafted with L1210 leukaemia and not treated, or treated by BCG (first injection 14 days before the graft and injections repeated every fourth day) or irradiated leukaemic cells (one injection 14 days before the graft), or association of both.

FIG. 3.—Tumour volume and cumulative survival of mice grafted with L1210 leukaemia and not treated or treated by BCG (first injection 24 hours after the graft and injections repeated each 4 days), or irradiated leukaemic cells (one injection 24 hours after the graft), or association of both.

FIG. 4.—Tumour volume and cumulative survival of mice grafted with L1210 leukaemia and not treated, or treated by BCG (first injection 4 days after the graft and injections repeated each 4 days), or irradiated leukaemic cells (one injection 4 days after the graft), or association of both.

FIG. 5.—Tumour volume and cumulative survival of mice grafted with L1210 leukaemia and not treated, or treated by BCG (first injection 6 days after the graft and injections repeated each 4 days), or irradiated leukaemic cells (one injection 6 days after the graft), or association of both.

FIG. 6.—Cumulative survival of mice grafted with $10^2$ to $10^7$ L1210 leukaemia cells: not treated or treated in the 24 hours following the graft by BCG (repeated injection) or irradiated leukaemic cells (one injection), or association of BCG and leukaemic cells.

$10^3$ leukaemic cells; the animals who only received $10^2$ cells did not have greater than 60 per cent mortality.

None of the three treatments was effective in the animals that received $10^7$ or $10^6$ leukaemic cells; on the other hand, BCG and the vaccination with irradiated tumour cells and the combination of these two treatments gave only an increase of the mean survival time and a cure of a certain number of the animals in the 4 groups of mice which had received $10^5$ leukaemic cells or less.

Table I shows a resumé of these results and a statistical analysis.

TABLE I.—*Experiment 3. Statistical Analysis of the Difference of Survival of Treated Group of Animals Compared to the Controls (P Values)*

| Number of Leukaemic cells grafted to induce the Leukaemias | $10^7$ | $10^6$ | $10^5$ | $10^4$ | $10^3$ | $10^2$ |
|---|---|---|---|---|---|---|
| BCG | 0 | 0 | 0·1 | 0·1 | 0·1 | 0·1 |
| Irradiated leukaemic cells | 0 | 0 | 0·1 | 0·1 | 0·1 | 0·1 |
| BCG + Irradiated leukaemic cells | 0 | 0 | 0·1 | 0·1 | 0·1 | NS |

This experiment also shows that the combination of repeated BCG and the administration of a single injection of irradiated leukaemic cells was more effective against the leukaemia than the administration of BCG alone, or of irradiated leukaemic cells, when the number of grafted leukaemic cells was $10^5$. This difference was very significant at the 30th day: $P < 1$ per cent.

### DISCUSSION

It has been shown that the administration, *before* the graft of a tumour, of adjuvants (Amiel, 1967; Biozzi *et al.*, 1959; Old, Clarke and Benacerraf, 1959) or of inactivated tumour cells (Glynn *et al.*, 1963), can inhibit the growth of the tumour. Active immunotherapy was only conceived as a method of preventive therapy, such as in the case of infectious diseases. But *preventive* active immunotherapy is outside any therapeutic, clinical application, because of the ignorance of tumour antigens in human cancers.

The experiments reported in this work demonstrate the possible *curative* action of active immunotherapy against an established experimental tumour.

The L1210 leukaemia was grafted into $F_1$ hybrid animals and makes it likely that allogeneic inhibition effects (Moller, 1964) were probably associated with the inhibition caused by the immunisation. Choquet and Malaise (unpublished data), working in this laboratory, have shown that allogeneic inhibition is certainly present when L1210 leukaemic cells are grafted into hybrid (C57BL/6 $\times$ DBA/2)$F_1$ mice, but the degree of inhibition is minor in relation to that caused by the immunisation. The control animals in our experiments enabled us to avoid attributing to immunisation any effect that may have arisen from allogeneic inhibition.

L1210 leukaemia is a leukaemia which has passed hosts by successive grafting and it can be questioned whether the leukaemic cells might have lost their tumour antigens; a study by Motta (1969) has shown that, though these antigens are feeble, they are still present in the cells.

Finally, it may be questioned if the immune effects obtained arise from these tumour antigens or from histocompatibility antigens bound to mutations sustained either by the leukaemia or by the DBA/2 line which forms part of the constitution of the $F_1$ hybrid. One can reply that, if histocompatibility antigens exist on the leukaemic cells that are not carried by the recipient mice, they are certainly very weak, for all grafts of this leukaemia kill 100 per cent of the recipients when $10^3$ or more tumour cells are given, and the animals are killed within 11 days once $10^6$ cells have been injected. Hence, if histocompatibility antigens do exist, then they can only act as very feeble antigens, with a force not greater than that of the tumour antigens. It is reasonable to hope that the conclusions reached in the present study can be applied to leukaemias whose only antigenic difference compared to the host, is due to their tumour antigens.

The first major concept that comes from these experiments is the power of active immunotherapy to eradicate leukaemia. Fig. 3 shows that 50 per cent of the mice treated with a combination of BCG and irradiated leukaemic cells were cured, and Fig. 7, which gives an overall analysis of an experiment and shows the growth of the tumour volume in each mouse, demonstrates that this therapy is also able to cause the regression of an established tumour. Though many chemotherapeutic drugs are capable of delaying the mortality of mice carrying L1210 leukaemia (Mathé, Schwarzenberg *et al.*, 1968), few are capable of curing 50 per cent of the treated animals, and causing the volume of an established tumour to regress, in the manner that was achieved with active immunotherapy in these experiments.

This power to eradicate leukaemia and to effect a cure, suggests that active immunotherapy is capable of destroying all the cells in a cancer population. This

**Fig. 7.**—Tumoral volume of each mouse in a group of mice carrying L1210 leukaemia and treated by the association of BCG and irradiated leukaemic cells 24 hours after the graft.

is in contrast to chemotherapy, which obeys first-order kinetics (Skipper, Schabel and Wilcox, 1964).

Two reservations to these concepts should be considered at this point: (a) in two mice out of 150 animals which survived to the 60th day after a graft of leukaemia, very late relapses occurred after 120 and 180 days respectively (these 150 animals were in part from the experiments described above, as well as from other experiments); these two relapses occurred in animals that were thought to have been cured; (b) though immunotherapy is capable of eradicating an entire tumour population in a certain percentage of the animals that have been treated, it is only capable of doing this if the population is relatively small. In the case of L1210 leukaemia in the mouse, only when the number of tumour cells in not greater than $10^5$. Skipper and his colleagues (1964) have shown that mice carrying L1210 leukaemia, and cured by the administration of cyclophosphamide, are insensitive to a new graft of this leukaemia, owing to an immunisation against the graft, but this only occurred when the number of tumour cells inoculated was not greater than $10^5$.

It will be seen that the pattern of tumour growth was according to a Gompertz function, consisting of two phases, rapid, then slow, and immunotherapy only acted upon the slow phase: (1) in some instances, immunotherapy reduces the slope of this part of the growth phase; in this case, it only slightly delays the mortality; (2) in other animals, the slope of this second phase descends, the tumour regresses and the animal is cured; (3) finally, in other animals, this slope is replaced by a plateau, associated with a considerable delay of the mortality, sometimes to as long as a month, which is very considerable for L1210 leukaemia: this observation, as well as the two very late relapses mentioned above, poses the question whether active immunotherapy may be capable of arresting and maintaining cells at Go for a long period; nevertheless, a study in progress, which is based on the cytophotometric examination of the cells of tumours the growth of which is represented by a plateau, suggests that, on the contrary, it acts essentially on the cell loss coefficient, it does not prolong the cycle, and it does not increase the percentage of cells in Go (L'heritier, Bulens and Speelman, 1969, personal communication).

From a practical point of view, it is convenient to stress that the repetition of the administration of BCG, the best of the adjuvants in this study, has a better curative effect than giving a single dose (but there is a slight increase in the mortality), whilst a single injection of irradiated cells is just as effective as repeated injections. BCG is more effective than irradiated cells when its administration is commenced before the graft of the leukaemia. A single injection of irradiated cells is more effective when given after the graft of the tumour than beforehand. But, in both these instances, there is another important, practical conclusion: the combination of BCG and the irradiated leukaemic cells is more effective at any of the time periods than when these stimuli are given alone (Fig. 2, 3, 4, 5).

This signifies that neither of these two treatments can induce a maximal stimulatory effect and that it is likely that they act upon two different systems. This has led us to suggest recommending in clinical practice the use of the combination of an adjuvant with a specific vaccine.

Mathé and his colleagues (Mathé, Amiel *et al.*, 1968; Mathé, *et al.*, 1969) have recently published the results of a clinical trial in which immunotherapy was used. The number of leukaemic cells was reduced to a level as low as possible, at first by chemotherapy to induce a remission, then by complementary chemotherapy, using sequentially all the drugs that are known to be effective against acute lymphoblastic leukaemia. The results of this clinical trial, which have been based upon the results of the experiments discussed in this paper, have been encouraging. Ten control patients treated by the chemotherapeutic regime and then receiving no further therapy, all relapsed within 130 days, following the cessation of chemotherapy; 12 patients of the 20 treated by active immunotherapy, consisting of either the application of BCG, or vaccination with irradiated leukaemic cells, or a combination of these two treatments, had not relapsed by 130 days. Seven of these patients have still not relapsed up to the present day: for one of them, this is more than $3\frac{1}{2}$ years; for 2, a period of more than $2\frac{1}{2}$ years, and for 4 others, periods of more than $1\frac{1}{2}$ years, since stopping chemotherapy.

### SUMMARY

(1) In the first experiment, a comparison was made of the effects of BCG, *Corynebacterium parvum*, *Mycobacterium cheiloni*, *Bordetella pertussis* or irradiated leukaemic cells, administered once or several times *after* the graft of $10^4$ L1210 leukaemic cells. Of the adjuvants, BCG was the only one with any notable effect, and repeated administration was more active than when given as a single dose; the irradiated leukaemic cells were more active than BCG, and had identical activity whether they were injected once or repeatedly.

(2) In the second experiment, a comparison was made of the effects of BCG, of irradiated leukaemic cells and a combination of them both, administered at various times in relation to a graft of $10^4$ L 1210 leukaemic cells. The BCG was given as repeated doses, whilst the irradiated leukaemic cells were given as a single dose. The BCG was more effective than the irradiated leukaemic cells when they were administered before the graft of the leukaemia; the irradiated leukaemic cells were more effective when they were given after the graft of the leukaemia. A combination of the two forms of immunotherapy was more effective than BCG alone, even when this was administered before the graft of the leukaemia, and more active than the irradiated leukaemic cells, even when they were administered after the graft of the leukaemia.

(3) In the third experiment, (C57BL/6 $\times$ DBA/2)$F_1$ mice were grafted with a variable number of L1210 (DBA/2) leukaemic cells. They were then treated during the 24 hours which followed this graft by active immunotherapy, either non-specific, using BCG, or specific, using leukaemic cells that had been irradiated at 15,000 rads, or by a combination of both. The three procedures were confirmed not only to be capable of prolonging the survival of the mice but also curing a considerable number of them. But cure was only obtained in those groups of animals in which the number of grafted cells was $10^5$ or fewer. When larger numbers of leukaemic cells were grafted the treatment was ineffective.

(4) The possible clinical application of these findings is discussed. They have already been applied to a trial of active immunotherapy for the treatment of acute lymphoblastic leukaemia in man, and have shown that such a therapy can be effective.

This work was supported by a grant from I.N.S.E.R.M., No. 66–235.

### REFERENCES

AMIEL, J. L.—(1967) *Revue fr. Étud. clin. biol.*, **12**, 912.

BALNER, H., OLD, L. J. AND CLARKE, D. A.—(1962) *Proc. Soc. exp. Biol. Med.*, **109**, 58.

BIOZZI, G., STIFFEL, C., HALPERN, B. N. AND MOUTON, D.—(1959) *C.r. Séanc. Soc. Biol.*, **153**, 987.

GLYNN, J. P., HUMPHREYS, S. R., TRIVERS, G., BIANCO, A. R. AND GOLDIN, A.—(1963) *Cancer Res.*, **23**, 1008.

HALPERN, B. N., BIOZZI, G., STIFFEL, C. AND MOUTON, D.—(1959) *C.r. Séanc. Soc. Biol.*, **153**, 919.—(1966) *Nature, Lond.*, **212**, 853.

HALPERN, B. N., PRÉVOT, A. R., BIOZZI, G., STIFFEL, C., MOUTON, D., MORARD, J. C., BOUTHILLIER, Y. AND DECREUSEFOND, C.—(1964) *J. reticuloendoth. Soc.*, **1**, 77.

MATHÉ, G.—(1968) *Revue fr. Étud. clin. biol.*, **13**, 881.

MATHÉ, G., AMIEL, J. L., SCHWARZENBERG, L., SCHNEIDER, M., CATTAN, A., SCHLUMBERGER, J. R., HAYAT, M. AND DE VASSAL, F.—(1968) *Revue fr. Étud. clin. biol.*, **13**, 454—(1969) *Lancet*, i, 697.

MATHÉ, G., SCHWARZENBERG, L., HAYAT, M. AND SCHNEIDER, M.—(1968) *Revue fr. Étud. clin. biol.*, **13**, 951.

MOLLER, G.—(1964) *Transplantation*, **2**, 405.

MOTTA, R.—(1969) *Immunology* (in press).

OLD, L. J., CLARKE, D. A. AND BENACERRAF, B.—(1959) *Nature, Lond.*, **184**, 291.

SKIPPER, E., SCHABEL, F. M. AND WILCOX, W. S.—(1964) *Cancer Chemother. Rep.*, **35**, 3.

WOODRUFF, M. F. A. AND BOAK, J. L.—(1966) *Br. J. Cancer*, **20**, 345.